# Sleep deprivation in dementia comorbidities: focus on cardiovascular disease, diabetes, anxiety/depression and thyroid disorders

**DOI:** 10.18632/aging.206157

**Published:** 2024-11-20

**Authors:** Upasana Mukherjee, Ujala Sehar, Malcolm Brownell, P. Hemachandra Reddy

**Affiliations:** 1 Department of Internal Medicine, Texas Tech University Health Sciences Center, Lubbock, TX 79430, USA; 2 Department of Nutritional Sciences, College Human Sciences, Texas Tech University, Lubbock, TX 79415, USA; 3 Department of Pharmacology and Neuroscience, Texas Tech University Health Sciences Center, Lubbock, TX 79430, USA; 4 Department of Neurology, Texas Tech University Health Sciences Center, Lubbock, TX 79430, USA; 5 Department of Public Health, Graduate School of Biomedical Sciences, Texas Tech University Health Sciences Center, Lubbock, TX 79430, USA; 6 Department of Speech, Language, and Hearing Sciences, Texas Tech University Health Sciences Center, Lubbock, TX 79430, USA

**Keywords:** dementia and comorbidities, sleep disturbances, cardiovascular disease, diabetes, thyroid disorders, anxiety, insomnia, sleep apnea

## Abstract

Sleep disturbances are a significant concern in individuals with dementia, affecting their overall health and quality of life, as well as that of their family members and caregivers. Dementia, a progressive neurodegenerative condition marked by cognitive decline, often coexists with various comorbidities such as cardiovascular disease, diabetes, obesity, anxiety/depression and thyroid disorders. These comorbidities can further impair cognitive function and complicate the clinical management of dementia, making it essential to address them in a holistic manner. This review critically examines the complex interplay between dementia and its associated comorbidities, with a special focus on the prevalence and impact of sleep disturbances. Sleep problems in dementia patients are not only common but also contribute to a faster progression of cognitive decline and increased burden on caregivers. The article explores the mechanisms by which these comorbidities, including cardiovascular conditions and metabolic disorders, exacerbate sleep disturbances and cognitive impairment in dementia patients. By synthesizing recent research findings, the review highlights the importance of identifying and managing modifiable risk factors for sleep disturbances in dementia. Integrated treatment approaches that address both cognitive and sleep-related challenges are essential for improving patient outcomes. The review also underscores the need for further research to develop targeted interventions that can effectively manage sleep disturbances in dementia, thereby enhancing the quality of life for both patients and caregivers. Understanding the relationship between dementia, comorbidities, and sleep disturbances is crucial for the development of comprehensive care strategies. This review aims to inform healthcare professionals about the current state of knowledge and encourage the implementation of evidence-based practices in dementia care.

## INTRODUCTION

Dementia is a general term used to describe a decline in cognitive function severe enough to interfere with daily life. It is not a specific disease but rather a group of symptoms characterized by a decline in memory, thinking skills, reasoning, judgment, and communication abilities. These changes may also affect a person’s behavior and ability to perform everyday tasks. Symptoms of dementia can vary widely depending on the type and stage of the condition but commonly include memory loss, especially recent memory, difficulty communicating or finding words, trouble with complex tasks such as managing finances or planning meals, confusion about time or place, changes in mood, personality, or behavior, withdrawal from social activities and interests [1]. Diagnosis typically involves a comprehensive assessment by a healthcare professional, including medical history, physical examination, neurological tests, and cognitive assessments. Imaging studies such as MRI or CT scans may also be used to rule out other causes of symptoms [2]. While there is no cure for most types of dementia, treatments and interventions can help manage symptoms, improve quality of life, and support caregivers [3]. These may include medications to temporarily improve cognitive function or manage behavioral symptoms, lifestyle modifications, cognitive therapies, and support services. Dementia is a progressive condition, meaning symptoms worsen over time, affecting the individual’s ability to function independently. However, early diagnosis and appropriate management can help individuals and their families navigate the challenges associated with dementia more effectively [4].

**Figure 1:**
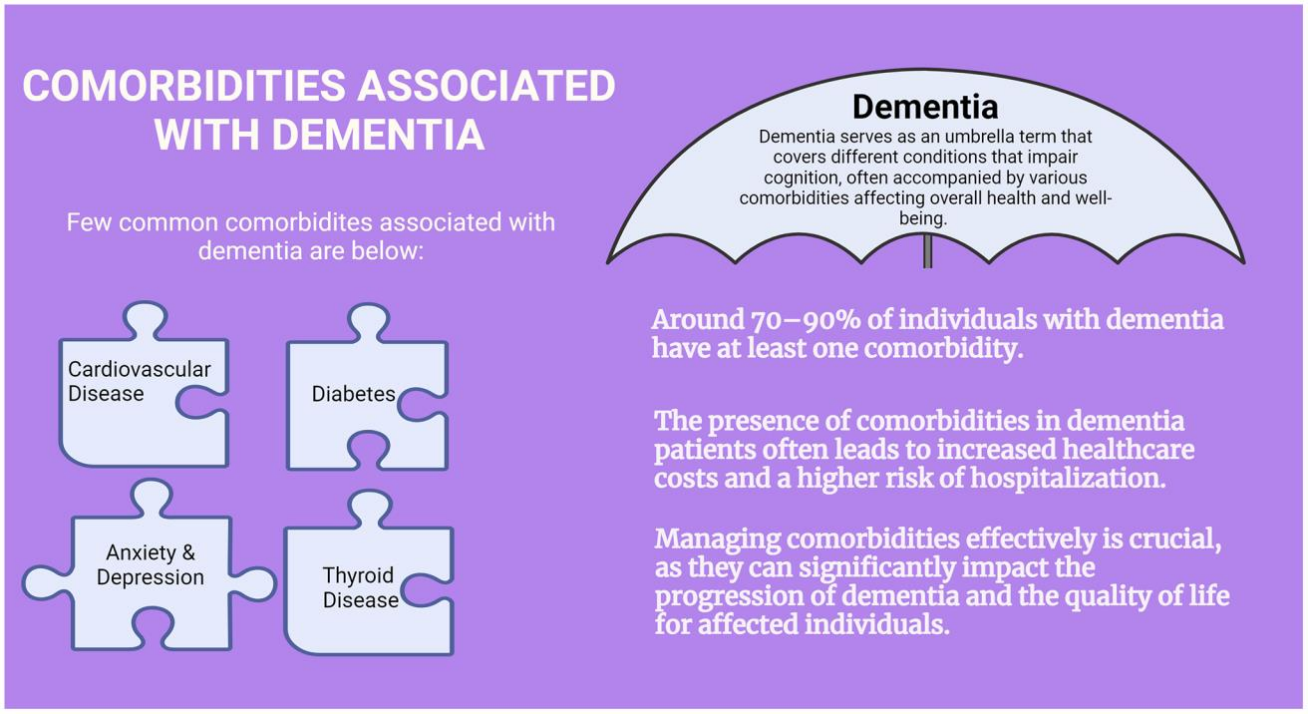
**Comorbidities associated with dementia.** Comorbidities associated with dementia, such as cardiovascular disease, diabetes, depression, and thyroid disease can significantly impact the progression and management of the condition. These additional health issues often exacerbate cognitive decline and complicate treatment strategies, highlighting the need for a comprehensive approach to care those addresses both dementia and its associated comorbidities. Understanding these relationships is crucial for improving patient outcomes and enhancing quality of life.

Dementia is often associated with several common comorbidities, which are other medical conditions that frequently occur alongside dementia (Figure 1). These comorbidities can complicate the management of dementia and impact overall health and quality of life. Conditions such as hypertension (high blood pressure), coronary artery disease, and atrial fibrillation are commonly seen in individuals with dementia. Diabetes mellitus, especially type 2 diabetes, is known to increase the risk of developing Alzheimer’s disease and vascular dementia [5]. Poorly managed diabetes can also contribute to cognitive decline and increase the severity of dementia symptoms. Mental health disorders, particularly depression and anxiety, frequently coexist with dementia. Depression can precede dementia or develop as a result of the challenges associated with cognitive decline and functional impairment. Disorders of the thyroid gland, such as hypothyroidism, can mimic symptoms of dementia or exacerbate cognitive impairment if left untreated. Regular thyroid function testing is important in individuals with dementia. Poor nutrition and unintended weight loss are common in dementia patients due to difficulties with eating, swallowing problems, and changes in appetite or taste perception. Malnutrition can worsen cognitive decline and overall health. Managing dementia involves addressing these comorbidities alongside cognitive and behavioral symptoms to improve overall quality of life. Careful monitoring, regular medical check-ups, and coordinated care between healthcare providers are essential in managing both dementia and its associated comorbidities effectively [6].

Sleep disturbances are a pervasive and challenging aspect of dementia, affecting a significant proportion of individuals living with these neurodegenerative conditions. As dementia progresses, disruptions in sleep patterns become increasingly common, presenting as insomnia, nocturnal wandering, daytime sleepiness, and other related issues [7]. These disturbances not only exacerbate the cognitive decline and behavioral symptoms associated with dementia but also significantly impact the quality of life for both patients and their caregivers. Understanding the prevalence and impact of sleep issues in dementia patients is crucial for providing comprehensive care and improving overall outcomes [8]. Research indicates that up to 50–70% of individuals with dementia experience sleep disturbances, far exceeding the prevalence in the general elderly population. These disturbances are multifaceted, influenced by neurobiological changes, comorbid medical conditions, environmental factors, and the complex interplay of dementia pathology itself [9]. Moreover, the consequences of poor sleep in dementia patients extend beyond mere discomfort. They contribute to increased caregiver stress, higher rates of institutionalization, and accelerated cognitive decline, further underscoring the urgent need for effective management strategies. By exploring the underlying mechanisms, prevalence rates, and broader implications of sleep disturbances in dementia, healthcare professionals can better tailor interventions to enhance sleep quality and overall well-being in this vulnerable population [10].

The purpose of this review article is to systematically examine the various causes and outcomes of dementia, with a particular focus on its correlation with other comorbidities. This review aims to synthesize current research findings to elucidate how underlying medical conditions, such as cardiovascular disease, diabetes, and thyroid disorders, influence the onset, progression, and severity of dementia. By analyzing these relationships, the article seeks to provide a comprehensive understanding of how comorbidities impact dementia risk and outcomes, identify potential modifiable risk factors, and inform strategies for integrated management and treatment approaches. This review article also aims to examine the prevalence, nature, and implications of sleep disturbances in patients with dementia and their comorbidities. By synthesizing current research on how sleep issues affect individuals with various types of dementia and associated conditions, this article seeks to provide a comprehensive understanding of the interplay between sleep and cognitive decline.

## Dementia and its types

The most common cause of dementia is Alzheimer’s disease, accounting for 60–80% of cases. Other causes include vascular dementia, which occurs after a stroke, Lewy body dementia, frontotemporal dementia, and others. Sometimes, individuals may have mixed dementia, where more than one type of dementia is present. Alzheimer’s disease, named after the German psychiatrist Alois Alzheimer who first described it over a century ago, is the most prevalent form of dementia, accounting for up to 75% of cases either alone or in conjunction with other pathologies, known as ‘mixed dementia’. Vascular dementia ranks second, arising from compromised brain blood supply due to arterial disease, leading to neuronal dysfunction and eventual cell death. Contributing factors include hypertension, hyperlipidemia, diabetes, smoking, diet, and obesity. Diabetes elevates dementia risk through both vascular complications and amylin-derived cerebral deposits. Vascular dementia can emerge post-stroke, typically progressing gradually rather than in discrete steps, manifesting in various ways depending on pathology location. Similar to Alzheimer’s disease, it involves memory and language impairments, alongside cognitive slowing, depression, anxiety, and apathy [11]. Dementia with Lewy bodies follows as the third most common type, constituting around 10% of cases and sharing characteristics with Alzheimer’s and Parkinson’s diseases. Lewy bodies, small protein aggregations of alpha-synuclein found throughout the brain, including the cerebral cortex, characterize this condition [12]. Clinical features encompass memory loss akin to Alzheimer’s, impaired alertness maintenance, spatial disorientation, and planning difficulties. Parkinson’s-like symptoms such as limb tremors, shuffling gait, and reduced facial expressions are also present. Unique attributes include visual hallucinations, recurrent falls, marked consciousness fluctuations, and sleep disturbances or nightmares. Frontotemporal dementia, comparatively rare, encompasses conditions affecting the frontal and temporal brain regions governing planning, emotion, motivation, and language. Types vary based on affected brain areas, with behavioral variant frontotemporal dementia featuring profound behavioral changes affecting personality, empathy, and mental flexibility. Changes in eating habits, such as overeating and a preference for sweets, are common. Primary progressive aphasia, another form, involves speech and language impairments like difficulty speaking or understanding words and concepts (semantic dementia) [13]. Mixed dementia refers to the coexistence of multiple dementia types, most frequently Alzheimer’s and vascular dementias, especially prevalent beyond the age of 80. Post-mortem examinations often reveal concurrent Alzheimer’s and vascular pathologies. This condition typically manifests as a gradual decline similar to Alzheimer’s disease but with added contributions from mini-strokes or strokes. Individuals often exhibit a history of vascular diseases or risk factors like ischemic heart disease, hypertension, diabetes, high lipid levels, or smoking [14, 15].

Huntington’s disease is an inherited disorder characterized by movement issues and cognitive decline, with dementia affecting about half of advanced cases. Corticobasal degeneration involves brain damage and protein deposits, causing movement problems and dementia [16]. Creutzfeldt-Jacob disease, caused by infectious prions, results in mood changes, memory lapses, and rapid progression to dementia and death. Multiple sclerosis can lead to cognitive difficulties if brain damage occurs, while normal pressure hydrocephalus causes balance issues, incontinence, and cognitive problems due to brain fluid accumulation. HIV-related dementia results from direct brain infection or immune deficiency, persisting despite antiretroviral treatments [17, 18].

## Comorbidities associated with dementia

### Cardiovascular diseases

Cardiovascular diseases (CVDs) like myocardial infarction (MI), heart failure (HF), and atrial fibrillation (AF) are known predictors of transient ischemic attacks (TIA) or stroke, which can cause cognitive impairment. MI is linked to vascular dementia (VD) through shared risk factors and systemic atherosclerotic disease. HF and AF, in particular, have a stronger association with incident dementia, including VD, potentially due to repeated cerebral microembolization and chronic hypoperfusion. Oral anticoagulation in AF may help prevent dementia, and irregular heartbeat in AF could impair cerebral regulation. HF is also related to Alzheimer’s disease, though this connection is less consistent across studies. Further research is needed to confirm these mechanisms and their impact on cognitive decline [19]. Cardiovascular risk factors can contribute to vascular dementia and may exacerbate symptoms of other types of dementia. Generalized atherosclerosis and its associated risk factors are crucial in the development of dementia, whereas reduced cardiac output appears to be less significant.

Researchers have proposed several mechanisms for this connection, such as ischemic lesions damaging specific brain networks or ischemia inducing inflammatory responses that lead to neuronal damage. Addressing and managing all modifiable risk factors for chronic vascular disease could, therefore, play a key role in lowering the rates of mild cognitive impairment and subsequent dementia [20]. A possible causal role for hypertension in the development of Alzheimer’s disease could be through the induction of atherosclerosis in cerebral arteries and by causing micro- and macro bleeds, white matter lesions, and micro infarcts. Thus, hypertension could be an important and treatable risk factor for Alzheimer’s disease. A reduced risk of Alzheimer’s disease was found for those taking diuretic antihypertensive medication compared with those not taking it. The use of antihypertensive medication was associated with a lower risk of Alzheimer’s disease [21]. Cardiovascular disease is linked to an increased risk of cognitive decline and dementia, but the extent of this risk across different cardiovascular conditions and in the absence of stroke is not well understood. Meta-analyses conducted to evaluate this risk focused on cardiovascular diagnoses (atrial fibrillation, congestive heart failure, peripheral artery disease, myocardial infarction) and on atherosclerotic burden (stenosis, calcification, and plaque characteristics) found that atrial fibrillation and severe atherosclerosis significantly increase the risk of cognitive decline or dementia. Though evidence for other cardiovascular conditions is less clear, atrial fibrillation and generalized atherosclerosis are identified as notable risk factors for cognitive deterioration [22].

Cerebrovascular lesions, such as lacunes and white matter ischemia, are prevalent in elderly patients with dementia, and vascular dementia (VaD) is the second most common form of dementia after Alzheimer’s disease (AD). Lacunar strokes are increasingly recognized as influential in AD’s clinical manifestation, and vascular risk factors are known to heighten the risk of developing AD. Stroke is a leading cause of death worldwide, with about 25–41% of stroke survivors over 65 developing VaD within three months. In the USA, approximately 125,000 new cases of VaD occur annually following ischemic stroke, representing a significant portion of dementia cases. This suggests that "mixed" dementia, involving both AD and cerebrovascular disease, may be more common than pure AD. Ischemic heart disease (IHD), which leads to congestive heart failure (CHF), is expected to be the leading cause of disability globally by 2020 [19, 21].

Cognitive impairment affects 26% of CHF patients post-hospitalization, with symptoms linked to heart function and blood pressure. Additionally, cognitive dysfunction is prevalent after coronary artery bypass graft (CABG) surgery, with up to 90% of patients showing cognitive issues at discharge and a 42% long-term incidence. Postoperative cognitive dysfunction is also noted in 26% of elderly patients after major surgeries. Vascular dementia may be significantly underdiagnosed and undertreated in the elderly, with both stroke and ischemic heart disease playing major roles in its prevalence [23, 24]. Systematic reviews indicate that cardiovascular disease (CVD)—including coronary heart disease, heart failure, and atrial fibrillation—impairs cognitive function and increases the risk of dementia, including all-cause, Alzheimer’s, and vascular dementia. Vascular risk factors such as hypertension, hyperlipidemia, diabetes, obesity, metabolic syndrome, and physical inactivity, as well as CVD risk prediction models like the Framingham scores, are linked to cognitive decline and dementia in older adults. Cognitive deficits are primarily observed in attention, executive function, and processing speed. However, in individuals aged 85 and older, the impact of CVD and these risk factors on cognitive decline may be less pronounced or even protective. Conditions like obesity, hypertension, metabolic syndrome, and hypercholesterolemia may show reduced or protective effects in this very old age group. For example, obesity might support bodily functions in frail individuals, and higher blood pressure could maintain brain perfusion. The metabolic syndrome’s impact may be diminished due to its composite nature and age-insensitivity in measurements, while higher cholesterol could be beneficial due to its role in neuron function. Overall, CVD and its risk factors are significant for cognitive impairment in middle-aged and young-old adults but may have different effects in the very old. The timing of disease onset and sample characteristics are crucial in determining the associations between CVD, its risk factors, and cognitive function. Further research is needed to explore how the duration and timing of CVD impact cognitive outcomes throughout life [25]. Anxiety is also linked to cardiovascular issues, which are known risk factors for vascular dementia and cognitive decline. The physiological responses triggered by anxiety, such as increased heart rate, higher blood pressure, vasoconstriction, and heightened platelet activity, can contribute to cardiovascular diseases like coronary artery disease and stroke. These conditions can lead to reduced blood flow to the brain, resulting in damage to brain tissue and subsequent cognitive impairment. This pathway suggests that anxiety might indirectly cause cognitive decline by exacerbating cardiovascular conditions that affect brain health [26].

### Diabetes

Dementia and non-insulin-dependent diabetes mellitus (NIDDM) are both common among the elderly. While diabetes has been known to impact cognition, its relationship with dementia remains unclear. Diabetes was identified through anti-diabetes medication use or elevated serum glucose levels. Dementia diagnosis followed a thorough process including sensitive screening and detailed diagnostic procedures. Among the participants, 724 (11.4%) had diabetes, and 59 out of 265 dementia patients (22.3%) had diabetes. Multiple logistic regression, adjusted for age and sex, showed a positive association between diabetes and dementia (odds ratio: 1.3, 95% confidence interval: 1.0–1.9). Notably, diabetes treated with insulin had a stronger association with dementia (odds ratio: 3.2, 95% confidence interval: 1.4–7.5). This association was most pronounced with vascular dementia but also noted in Alzheimer’s disease. These findings remained significant after accounting for factors such as education, smoking, body mass index, atherosclerosis, blood pressure, and antihypertensive treatment, and were not explained by clinical cerebral infarctions. The results suggest that NIDDM is linked to an increased risk of dementia, particularly in elderly individuals with insulin-treated diabetes [27].

Recent research indicates that the molecular defects linked to diabetes also increase the risk of various types of dementia, including Alzheimer’s disease, vascular dementia, and Pick’s disease. Type II diabetes mellitus is associated with a two- to three-fold higher risk of developing dementia. With approximately 250 million people worldwide (over 2 million in the UK) currently diagnosed with diabetes, and this number expected to double in the next 20 years, the related risk could lead to a significant rise in dementia cases. The molecular connections between insulin action, diabetes, and Alzheimer’s disease, and potential therapeutic interventions focuses on the regulation of glycogen synthase kinase-3 (GSK-3) and impacts neuronal function [28]. Both obesity and diabetes are significant risk factors for developing dementia. Obesity contributes to cognitive decline through mechanisms like chronic inflammation, insulin resistance, and altered lipid metabolism. Similarly, diabetes increases dementia risk by promoting vascular damage and disrupting glucose metabolism in the brain. There is a need for effective management of obesity and diabetes to potentially reduce the risk of dementia and improve cognitive health [29]. Type 2 diabetes mellitus (DM) is linked to an increased risk of cognitive decline and dementia, including Alzheimer’s disease (AD) and vascular dementia (VaD).

A specific subtype of dementia, termed “diabetes-related dementia” (DrD), is associated with distinct DM-related metabolic abnormalities rather than AD pathology or cerebrovascular disease. DrD is characterized by advanced age, high hemoglobin A1c, prolonged diabetes duration, frequent insulin use, and minimal apolipoprotein E4 presence. It shows less severe medial temporal lobe atrophy and slower progression of cognitive impairment, with particular deficits in attention and executive function but less impairment in word recall. DrD often lacks significant amyloid accumulation seen in AD and is associated with insulin resistance, elevated inflammatory cytokines, oxidative stress, and advanced glycation end products. Glycemic control can improve cognitive functions like attention and executive skills in DrD patients. Additionally, DrD patients have higher rates of frailty and sarcopenia/dynapenia compared to AD patients, indicating a need for geriatric-focused interventions. Identifying DrD as a distinct type of dementia is important for developing appropriate therapies and prevention strategies [30]. Emerging epidemiological evidence indicates that individuals with diabetes mellitus face a heightened risk of developing dementia, although findings regarding specific dementia subtypes are inconsistent. Diabetes mellitus is associated with a 1.5- to 2.5-fold increased risk of dementia in elderly community-dwelling individuals, affecting both vascular dementia and Alzheimer’s disease. The exact mechanisms are not fully understood but may involve a combination of factors such as cardiovascular risks, glucose toxicity, altered insulin metabolism, and inflammation. Effective management of these risk factors early in life could be crucial in preventing dementia. Additionally, innovative therapeutic approaches are needed to address and mitigate the risk of dementia in people with diabetes [31].

Research has established an epidemiological link between diabetes mellitus (DM) and dementia, with a clearer association observed for vascular dementia, though it is also seen with Alzheimer’s disease. There are various hypotheses explaining the connection between DM and dementia. Potential mechanisms include acute hyperglycemia, chronic hyperglycemia-induced microangiopathy, hypoglycemia, and insulin resistance. The concept of "type 3 diabetes" suggests that insulin resistance leads to reduced brain insulin, impaired regulation of insulin-degrading enzyme, and beta-amyloid accumulation, which could contribute to AD. Improved metabolic control, particularly in middle age, is associated with better cognitive performance. However, there is no definitive evidence that any specific class of diabetes medication is superior. Clinicians managing diabetes should be aware of its potential link to dementia and incorporate cognitive assessments into routine care [32].

### Anxiety and depression

Studying anxiety in dementia poses challenges due to overlapping symptoms between the two conditions. Generalized Anxiety Disorder (GAD) symptoms such as restlessness, fatigue, and concentration difficulties can also occur in dementia, making it hard to diagnose anxiety disorders reliably, especially in individuals with language impairments. The DSM-IV suggests evaluating whether symptoms can be attributed to the physiological effects of dementia to determine if they signify an independent anxiety disorder. Several strategies have been proposed to address this issue. Some studies use standard DSM-IV criteria for diagnosing anxiety without considering the overlap with dementia symptoms, potentially leading to overestimation of anxiety disorders. Tools designed to assess neuropsychiatric symptoms in dementia aim to minimize overlap with dementia symptoms and include items more indicative of anxiety, such as fear of being alone. Researchers have proposed revised GAD criteria for individuals with dementia, including excessive, uncontrollable worry and additional specific symptoms like restlessness and muscle tension. Among these strategies, using specialized instruments and revised diagnostic criteria seems most effective in accurately distinguishing anxiety from dementia symptoms. The revised criteria approach should be supported by expert consensus and empirical data to improve diagnostic accuracy and better address anxiety in dementia patients [33]. The validity of DSM-IV and ICD-10 criteria for diagnosing generalized anxiety disorder (GAD) in Alzheimer’s disease (AD), identification of symptoms associated with excessive anxiety and worry in AD, and assessment of the co-occurrence of GAD and depression, as well as the impact of GAD on cognitive and functional outcomes in AD is discussed here. Among 552 patients with probable AD, 26% reported excessive anxiety and worry. This anxiety was linked to symptoms such as restlessness, irritability, muscle tension, fears, and respiratory issues. Using revised criteria based on these symptoms, 10% of patients met the criteria for GAD, compared to 15% using DSM-IV criteria and 9% using ICD-10 criteria. GAD was present in 26% of patients with major depression and 5% of those without depression. Patients with both GAD and depression exhibited more severe cognitive impairments than those with only one of these conditions. The study validated a set of diagnostic criteria for GAD in dementia, emphasizing the frequent co-occurrence of GAD with major depression in AD patients and its exacerbating effect on cognitive deficits [34].

Depression and anxiety are prevalent among individuals with dementia and mild cognitive impairment (MCI), with rates varying widely. Depression affects 10% to 62% of those with dementia, with lower rates using stricter criteria, and 36% to 63% of those with MCI. Anxiety rates range from 8% to 71% in dementia patients and 10% to 74% in MCI patients. Anxiety-specific disorders can affect up to 49% of individuals with dementia. These symptoms significantly impact the ability to live independently, increase the risk of institutionalization, and add to caregiver burden. Depression and anxiety in MCI often resist antidepressant treatments and are linked to a higher risk of progression to Alzheimer’s disease. Despite recommendations to address these symptoms in dementia care, pharmacological treatments are often ineffective and have side effects [35]. The link between depression and the risk of developing dementia remains unclear, with varying study results contributing to the ambiguity. Research has explored different aspects, such as the relationship between the number of depressive symptoms and Alzheimer’s disease (AD) diagnosis, or comparing the risk of dementia between patients with unipolar or bipolar affective disorders and those with chronic conditions like diabetes. Some studies suggest that patients with mood disorders have a higher risk of developing dementia, while others report conflicting findings. Overall, both retrospective and prospective studies indicate that depression, including subsyndromic forms with depressive symptoms but not full-blown depression, may increase the risk of developing dementia. Persistent depressive symptoms, even if not meeting full diagnostic criteria, are associated with a higher likelihood of progressing to dementia. This risk appears to be significant even for individuals with only mild depressive symptoms [36].

**Figure 2:**
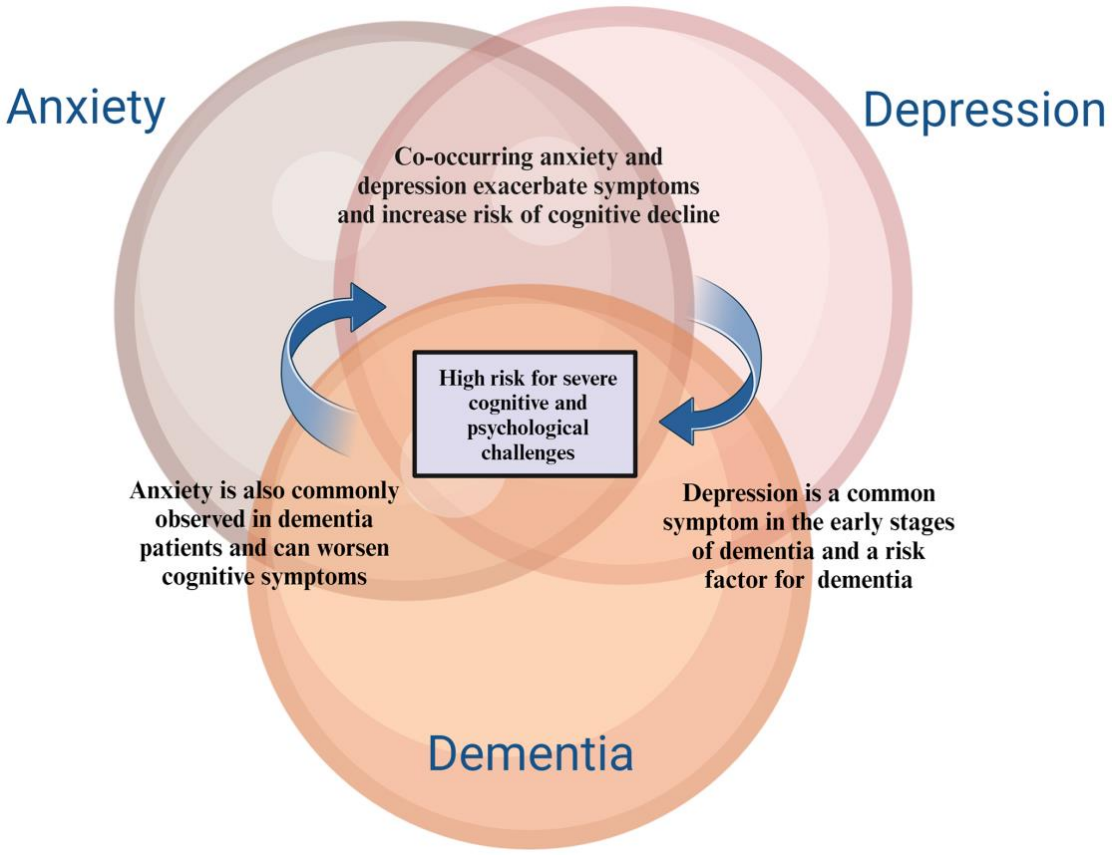
**Relationship between anxiety, depression and dementia.** The relationship between anxiety, depression, and dementia is complex and bidirectional. Both anxiety and depression are common in individuals with dementia, often exacerbating cognitive decline and worsening quality of life. Conversely, early signs of dementia can lead to increased feelings of anxiety and depression, creating a challenging cycle that necessitates integrated mental health support in dementia care.

The potential causal pathways linking anxiety to cognitive decline and dementia involve several biological and psychological mechanisms. One of the primary hypotheses is that anxiety leads to chronic stress, which, in turn, results in elevated levels of cortisol, a stress hormone. Prolonged exposure to high cortisol levels can have several detrimental effects on the brain, particularly in areas like the hippocampus, which is crucial for memory and learning (Figure 2). Chronic hypercortisolism can cause atrophy of the hippocampus, impairing its function and potentially accelerating cognitive decline. In animal studies, elevated cortisol has been shown to increase amyloid formation and tau accumulation, both hallmark features of Alzheimer’s disease. Chronic anxiety has been associated with increased levels of inflammatory cytokines, such as interleukin-6 and tumor necrosis factor. These cytokines are known to have negative effects on cognitive functioning. The theory posits that the chronic low-grade inflammation observed in anxiety disorders could contribute to neurodegenerative processes. Inflammation in the brain can lead to neuronal damage and impair synaptic function, both of which are critical for maintaining cognitive health. Over time, this inflammation could contribute to the development of conditions like Alzheimer’s disease [37]. BDNF is a protein that plays a crucial role in supporting the survival of existing neurons and encouraging the growth and differentiation of new neurons and synapses. It is essential for learning, memory, and overall brain plasticity. Anxiety disorders have been associated with decreased levels of BDNF, which could impair the brain’s ability to repair and maintain its neural networks. In patients with Alzheimer’s disease and mild cognitive impairment (MCI), reduced BDNF levels have been observed, suggesting that anxiety might contribute to cognitive decline by suppressing this critical neurotrophic factor. Cognitive reserve refers to the brain’s ability to improvise and find alternate ways of completing tasks despite damage. Individuals with higher cognitive reserves are better able to compensate for the early effects of neurodegenerative changes. Anxiety disorders, particularly those that are chronic and accompanied by avoidance behavior, can lead to decreased cognitive and social stimulation, reducing cognitive reserves. This reduction makes individuals more vulnerable to cognitive decline and dementia, as they have less cognitive capacity to buffer against the effects of brain aging and neurodegenerative diseases. Another possibility is that anxiety might not be a direct cause but rather a prodromal symptom of dementia. Older adults who are beginning to experience subtle cognitive decline may develop anxiety as they become aware of their cognitive difficulties and fear the onset of dementia. This anxiety could then exacerbate the cognitive decline, creating a vicious cycle. As the neurodegenerative process advances, anxiety levels might decrease due to the worsening of cognitive functions, which may impair the individual’s awareness of their condition [26, 38].

### Thyroid disorders

Elevated and high-normal thyroid function is linked to an increased risk of dementia. However, thyroid function does not correlate with vascular brain disease as evaluated by MRI, indicating that thyroid hormones may contribute to dementia through non-vascular mechanisms [39]. The link between thyroid function, cognitive status, and dementia severity, highlight the potential for thyroid dysfunction to influence dementia risk. Dementia, a debilitating condition that impairs independence and quality of life while straining healthcare systems, could potentially be mitigated by identifying modifiable risk factors. Thyroid hormones, crucial for central nervous system development, have been linked to Alzheimer’s disease (AD) and vascular dementia (VD), with both subclinical hyperthyroidism and hypothyroidism identified as risk factors for cognitive decline. While some studies propose a narrower target range for thyroid-stimulating hormone (TSH) to better manage these risks, others have found inconsistent results. For instance, research indicates a strong correlation between high thyroid dysfunction and dementia severity, aligning with studies suggesting that both elevated TSH and decreased FT3 levels are associated with worsened cognitive performance and progression from mild cognitive impairment to AD. Conversely, findings from other research have shown no significant relationship between thyroid dysfunction and dementia or have reported differing associations, such as high FT4 levels accelerating cognitive decline. Additionally, dementia prevalence increases with age and is more pronounced in females, which is consistent with some studies but contradicts others suggesting otherwise. These findings underscore the need for further investigation into thyroid function as a potential modifiable risk factor for dementia and suggest that tailored psychological and pharmacological interventions may be required to address this complex relationship effectively [40].

## The importance of sleep in dementia and comorbidities

**Figure 3:**
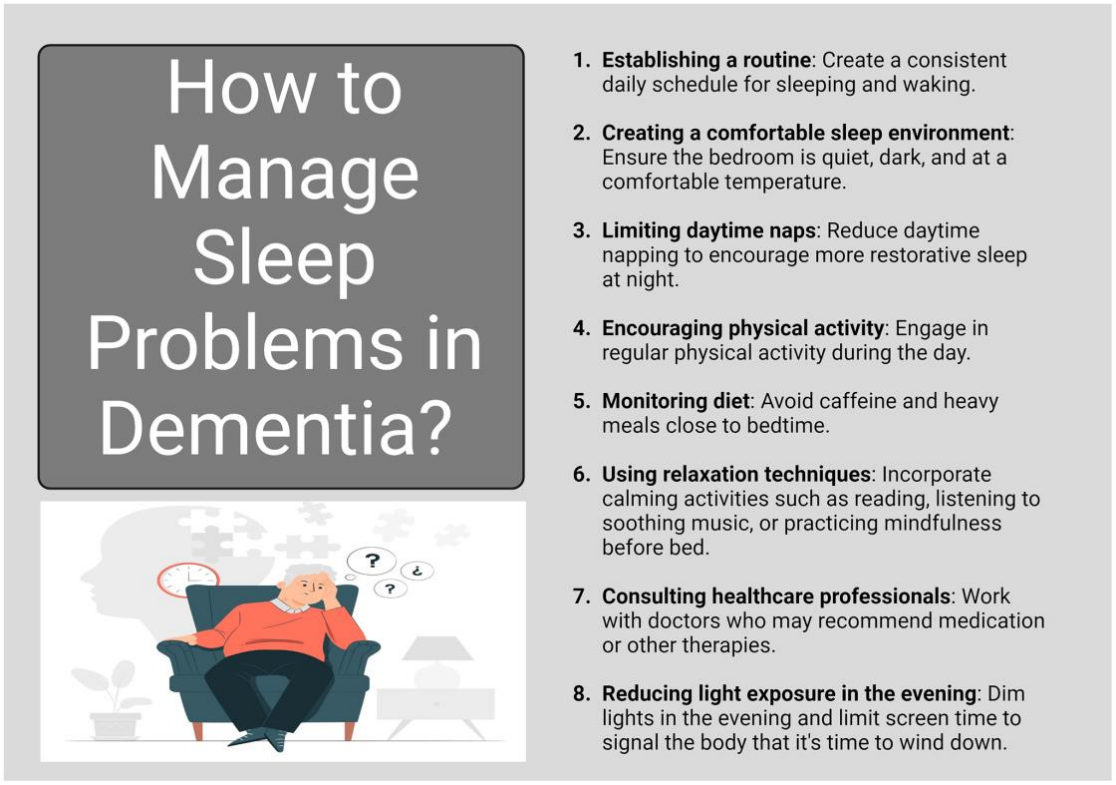
**Managing sleep problems in dementia.** Managing sleep problems in dementia is essential for improving overall well-being and cognitive function. Interventions may include creating a structured sleep routine, minimizing environmental disturbances, and considering behavioral therapies or medications when necessary. Addressing sleep issues can enhance daytime alertness and reduce the risk of agitation and confusion in individuals with dementia.

Sleep plays a crucial role in cognitive function and memory consolidation through several mechanisms. One of the primary functions of sleep is to consolidate memories formed during waking hours. This process involves transferring newly acquired information from short-term memory to long-term memory storage. During sleep, particularly during slow-wave sleep (SWS) and REM (rapid eye movement) sleep, the brain replays and strengthens neural connections associated with recent experiences and learning tasks. This consolidation process helps solidify memories, making them more stable and accessible. Sleep supports synaptic plasticity, which refers to the ability of synapses (connections between neurons) to strengthen or weaken over time in response to activity [41]. This plasticity is crucial for learning and memory formation. During sleep, the brain undergoes processes that promote synaptic consolidation, enhancing the retention of new information and skills learned during waking hours. Adequate sleep enhances cognitive functions such as attention, concentration, and problem-solving abilities [42]. These cognitive processes are essential for effective learning and academic or professional performance. Sleep deprivation, on the other hand, impairs these functions, making it more difficult to acquire and retain new information. Sleep helps organize and integrate memories by linking new information with existing knowledge and experiences stored in long-term memory. This process facilitates understanding and contextualization of information, promoting a deeper understanding of learned material [43]. Sleep plays a role in emotional regulation by processing and integrating emotional experiences and reactions. Adequate sleep supports resilience to stress and emotional stability, while sleep deprivation can lead to heightened emotional reactivity and difficulty in managing emotions. During sleep, the brain’s glymphatic system becomes more active, facilitating the clearance of metabolic waste products that accumulate during wakefulness. This detoxification process helps maintain optimal brain function and supports cognitive processes, including memory consolidation [44]. Overall, sleep is essential for maintaining optimal cognitive function, enhancing memory consolidation, and supporting overall brain health. Establishing good sleep hygiene practices and addressing sleep disorders promptly are crucial for preserving cognitive abilities and promoting lifelong learning and memory retention (Figure 3) [45].

Poor sleep quality is linked to increased anxiety, depression, and pain among healthy individuals. Poor sleep quality may contribute to fatigue, which in turn can exacerbate feelings of depression and pain. These factors together can negatively impact quality of life. The research highlights a significant connection between poor sleep and reduced emotional and physical well-being, suggesting that poor sleep quality affects various aspects of quality of life. However, the further research is necessary to better understand the complex relationships and causative factors between sleep quality, pain, and overall quality of life [46]. Sleep quality is the most influential factor affecting the five domains of quality of life - well-being, life satisfaction, subjective health, work stress, and happiness. Individuals with better sleep quality reported higher quality of life. Improvements in sleep quality over time is also associated with better quality of life. However, the quality of sleep is more important to quality of life than sleep duration or variations in sleep patterns between workdays and weekends [47, 48].

Psychosocial factors like stress, depression, and interpersonal relationships in both sleep quality and CVD risk. Racial/ethnic minorities in the U.S. experience significant disparities in sleep compared to non-Hispanic Whites, which may contribute to higher CVD rates in these groups (Hall et al., 2018). Individuals with short sleep duration have a higher risk of cardiovascular disease (CVD) and coronary heart disease (CHD) compared to normal sleepers, with risks increasing by 15% for total CVD and 23% for CHD. The risk is even higher—63% for CVD and 79% for CHD—among those with both short sleep duration and poor sleep quality. Long sleep duration is not associated with increased CVD or CHD risk and might even offer some protection against CHD. Poor sleep quality intensifies the risk of CVD and CHD in short sleepers, and it is important to consider both sleep duration and quality in cardiovascular health research [49]. Inadequate or disrupted sleep can lead to increased sympathetic nervous system activity, higher blood pressure, and elevated heart rate, all of which contribute to cardiovascular strain. Additionally, poor sleep is associated with metabolic dysregulation, including insulin resistance, increased inflammation, and adverse changes in lipid profiles. These physiological responses can promote the development of atherosclerosis, hypertension, and other cardiovascular conditions [50].

## Types of sleep disorders common in dementia and comorbidities

### Insomnia

**Figure 4:**
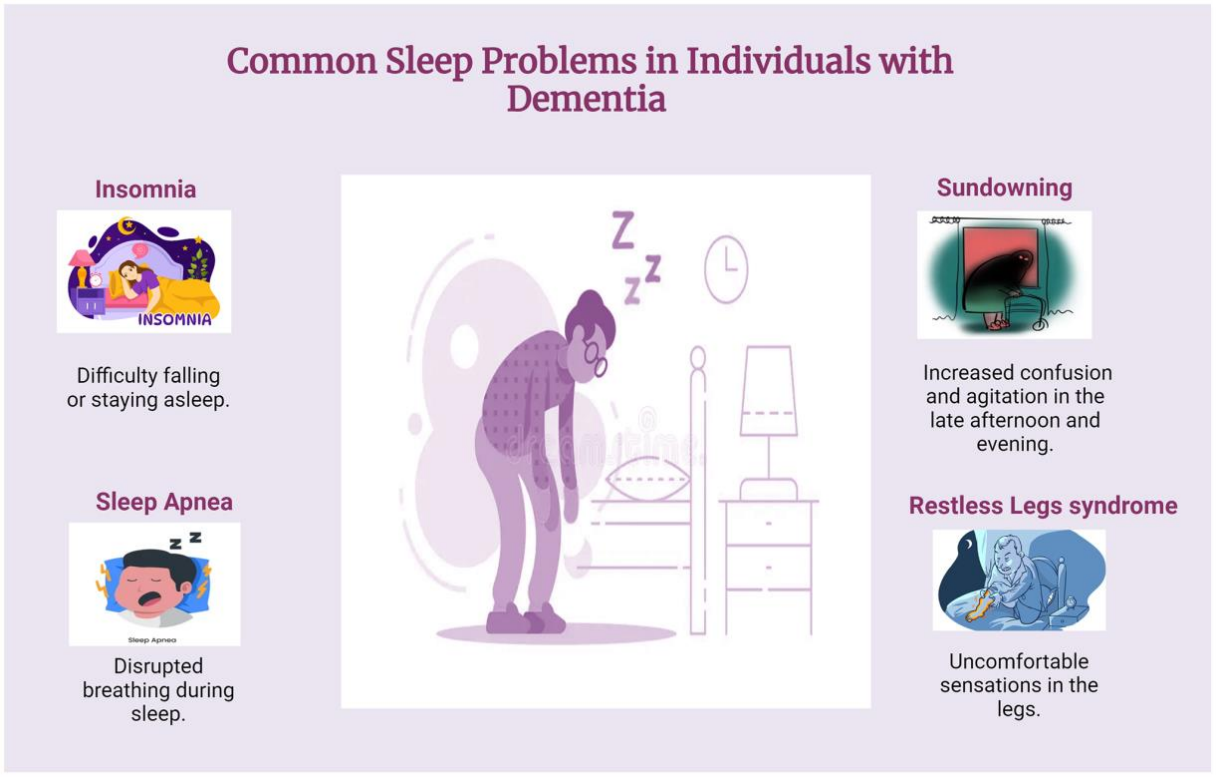
**Common sleep problems in dementia.** Common sleep problems in dementia include insomnia, fragmented sleep, sleep apnea, sundowning, and restless legs syndrome. Individuals may have trouble falling asleep or staying asleep due to altered circadian rhythms and increased confusion during the night. These sleep disturbances can exacerbate cognitive decline and contribute to behavioral issues, making effective management crucial for enhancing quality of life.

Insomnia disorder is defined by dissatisfaction with sleep quality or quantity, characterized by difficulty falling asleep, frequent nighttime awakenings with trouble returning to sleep, or waking up earlier than desired. It is associated with significant distress and impairment in daily functioning, including fatigue, daytime sleepiness, cognitive deficits, and mood disturbances (Figure 4). Unlike sleep deprivation, insomnia persists despite adequate opportunity for sleep [51]. Prevalence estimates vary, with 5% to 15% of individuals affected by insomnia disorder, and chronic insomnia affecting 31% to 75% of patients, lasting more than a year for the majority. Despite advancements in understanding its nature and causes, there remains no universally accepted model of insomnia, partly due to its diverse symptoms, high comorbidity rates, and varying levels of analysis from phenomenology to physiology [52]. A comprehensive model should explain symptom heterogeneity, the increased risk for comorbid conditions like depression and cardiometabolic syndrome, and discrepancies between self-reported and objectively measured symptoms. Understanding insomnia’s pathophysiology could inform prevention and treatment strategies [53]. Insomnia is often characterized as a disorder involving hyperarousal, encompassing increased somatic, cognitive, and cortical activation. Physiological hyperarousal affects both the central (cortical) and peripheral (autonomic) nervous systems in individuals with insomnia. Additionally, cognitive and emotional hyperarousal at bedtime is theorized to contribute to both acute and chronic forms of the disorder. Despite its frequent mention in literature, hyperarousal lacks a standardized definition. Hyperarousal is conceptualized as heightened physiological, emotional, or cognitive activity that disrupts the natural disengagement from the environment necessary for sleep onset. Various methods, including cortisol levels, heart rate variability, EEG measurements, and self-reported experiences, can indicate hyperarousal [54]. However, defining a precise threshold for hyperarousal remains challenging, leading studies to often rely on observed differences between insomnia and control groups rather than absolute criteria [55].

Insomnia is a common issue in clinical settings, occurring either on its own or alongside other medical or psychiatric disorders, and often requires specific treatment. Among the available therapies, benzodiazepine-receptor agonists (BzRAs) and cognitive-behavioral therapy (CBT) have the strongest empirical support. BzRAs are effective for short-term insomnia management and are easily accessible, but their long-term efficacy is limited and they carry potential side effects (Morin & Benca, 2012). CBT, while more time-consuming, is a highly effective treatment for chronic insomnia, offering lasting improvements and high patient acceptance. Although CBT is not widely available in most clinical settings, its delivery can be enhanced through innovative methods like telephone consultations, group therapy, and self-help strategies. Combining CBT with medication can improve outcomes, though there is limited evidence on the optimal integration of these treatments in clinical practice [56, 57].

Longitudinal studies have shown that individuals with insomnia disorder are more susceptible to developing psychopathological symptoms, particularly depression, compared to those who sleep well. Although previous research has established relatively stable effects, the most recent meta-analysis on this topic was published four years ago. To update this knowledge, a recent replication study was conducted, evaluating new research from 2018 to 2022 on the longitudinal relationship between insomnia disorder and psychopathology. The literature search, conducted between April 2018 and August 2022, identified only one new study relevant to the association between insomnia and depression. Meta-analytic results reinforced earlier findings, revealing an even stronger link between insomnia and depression, supporting the notion that insomnia may act as a transdiagnostic factor in psychopathology. Despite these insights, further longitudinal studies are needed to better understand the connection between insomnia disorder and various mental health disorders [57–59].

Studies across various countries, using both cross-sectional and longitudinal designs, consistently show that self-reported insomnia complaints are prevalent among older adults, with estimates ranging from 30% to 60%. Chronic insomnia is also common, affecting between 12% and 41% of this population. Insomnia can manifest as a symptom of another medical or psychiatric condition or as a disorder in its own right, which is heterogeneous in terms of duration, types, and underlying causes. Types include difficulty falling asleep, staying asleep, early morning awakening, or combinations thereof, often accompanied by nonrestorative sleep and perceived poor sleep quality despite adequate sleep opportunity [60]. Neurobiological studies have identified specific brain areas showing hypometabolism during wakefulness and increased metabolism during sleep in individuals with insomnia. These regions play crucial roles in arousal regulation, emotional processing, and cognitive function, providing a basis for understanding the frequent occurrence of sleep disturbances in psychiatric disorders and the associated health risks, such as impaired cognitive performance and increased susceptibility to conditions like depression and dementia. Insomnia may also be linked to neurodegenerative diseases, with disorders like REM sleep behavior disorder (RBD) occurring more frequently in synucleinopathies such as Parkinson’s disease and dementia with Lewy bodies compared to Alzheimer’s disease and frontotemporal dementia. Furthermore, insomnia is associated with cognitive impairment across various domains, including working memory and executive function, and is implicated in accelerated cognitive decline over time. However, the exact relationship between insomnia and dementia risk remains unclear, complicated by factors like long-term medication use and coexisting medical and psychiatric conditions that are both prevalent in insomnia patients and known risk factors for dementia [61, 62].

Patients with long-term insomnia using hypnotics face over a twofold increased risk of dementia, regardless of whether they use benzodiazepine or non-benzodiazepine medications. Higher prescribed dosages and longer half-life values of hypnotics further elevate this risk [63]. Notably, individuals aged 50–65 with long-term insomnia show a higher dementia risk compared to those over 65, possibly due to inherent age-related risks. The association between hypnotics and dementia risk may stem from their direct effects, exacerbating sleep disturbances in the prodromal phase of dementia or causing cognitive decline through mechanisms such as GABAergic transmission modulation and synaptic plasticity impairment. Despite conflicting evidence from previous studies on the relationship between sleep disturbance and dementia, daytime sleepiness rather than insomnia itself appears predictive. Thus, the significance of considering hypnotic use as a potential risk factor for dementia and the importance of cautious prescribing practices and early discontinuation strategies to mitigate cognitive risks associated with long-term hypnotic use is emphasized be clinicians [64, 65].

### Sleep apnea

**Figure 5:**
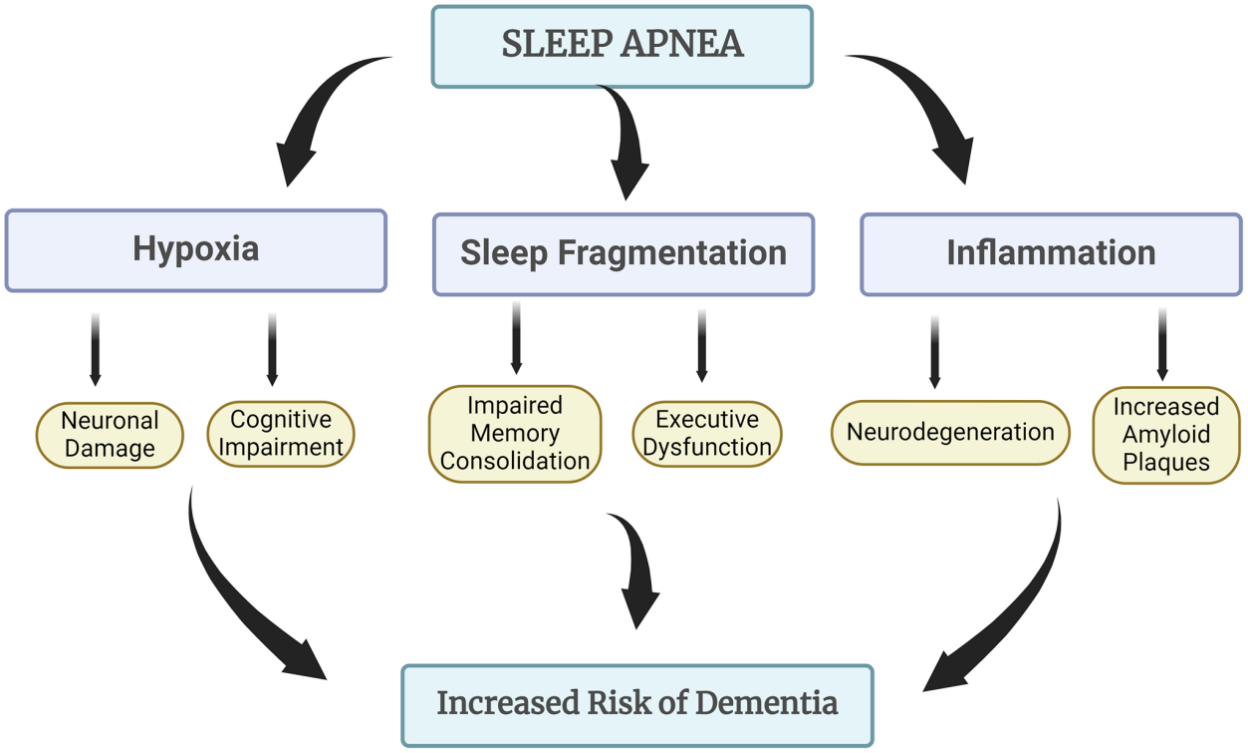
**How sleep apnea leads to increased risk of dementia.** Sleep apnea is associated with an increased risk of dementia due to its impact on oxygen levels during sleep and the subsequent stress it places on the brain. Repeated interruptions in breathing can lead to fragmented sleep and hypoxia, which may contribute to cognitive decline and neurodegeneration over time. Additionally, the exacerbation of inflammation linked to sleep apnea further heightens the risk of developing dementia-related conditions.

Sleep apnea is a serious sleep disorder characterized by repeated interruptions in breathing during sleep. These pauses in breathing can last from a few seconds to minutes and may occur hundreds of times throughout the night. Obstructive Sleep Apnea is the most common form, caused by a blockage of the upper airway, usually due to the relaxation of throat muscles and tissues. This blockage prevents airflow into the lungs despite the effort to breathe. Central Sleep Apnea occurs when the brain fails to send the proper signals to the muscles that control breathing. It is less common than OSA and is often associated with certain medical conditions, such as heart failure or stroke [66]. Complex Sleep Apnea Syndrome (Mixed Sleep Apnea) is a combination of obstructive and central sleep apnea. Common symptoms of sleep apnea include loud snoring, choking or gasping during sleep, excessive daytime sleepiness, difficulty concentrating, and irritability. If left untreated, sleep apnea can lead to serious health issues, including high blood pressure, heart disease, diabetes, and stroke. Diagnosis typically involves a sleep study, either at a sleep center or at home, to monitor breathing patterns and other vital signs during sleep. Treatment options vary depending on the severity of the condition and may include lifestyle changes, continuous positive airway pressure (CPAP) therapy, oral appliances, or surgery (Figure 5) [67].

Obstructive sleep apnea (OSA) and cardiovascular disease (CVD) frequently co-occur, suggesting shared risk factors and a bidirectional relationship. Multiple mechanisms indicate that OSA is a modifiable risk factor for CVD, but the strength of this association varies across subgroups defined by age, sex, diabetes, and symptoms like insomnia and sleepiness, especially when the apnea-hypopnea index (AHI) is used as the primary metric for OSA. Clinical trials on the impact of CPAP therapy on CVD outcomes have mostly yielded inconclusive results. These trials often use OSA definitions that overlook subgroup differences and face various methodological challenges. New research indicates that key mechanistic traits of OSA—such as upper airway collapsibility, loop gain, and respiratory arousal threshold—differ among clinical and population subgroups, even in individuals with similar AHI levels. These traits are associated with distinct patterns of hypoxia, cardiac autonomic response, and respiratory arousal intensity that contribute to CVD. Recognizing the heterogeneity of OSA, along with advancements in risk stratification tools, provides a foundation for developing better diagnostic methods and testing targeted interventions in subgroups most likely to benefit from treatment [68]. There is a direct association between the severity of obstructive sleep apnea (OSA) and major cardiovascular disease (CVD) risk factors, including hypertension, obesity, diabetes, hyperlipidemia, and physical inactivity. Other risk factors for OSA, such as increasing age, higher BMI, alcoholism, smoking, and measures of sleep disruption like the apnea duration and oxygen desaturation index. Severe OSA is strongly linked to excessive daytime sleepiness and significant oxygen desaturation during sleep.

Heart disease, the leading cause of death globally, is strongly influenced by primary risk factors like diabetes, lipid levels, coronary artery function, and kidney function. A data mining model using a clinical database showed accuracy in predicting cardiovascular risk. Traditional cardiovascular risk factors, while affecting both sexes, may be more potent in women. Conditions like gestational hypertension and preeclampsia are associated with higher rates of sleep-disordered breathing, highlighting the importance of cardiovascular risk assessment in women. Additionally, up to 63% of individuals with OSA may experience depressive symptoms, which can significantly impact their quality of life [69].

### Restless legs syndrome

Restless Legs Syndrome (RLS) is a neurological condition characterized by an uncontrollable urge to move the legs, typically due to uncomfortable sensations. These sensations often occur when at rest and are relieved by movement. The condition usually worsens in the evening or at night, leading to difficulties with falling or staying asleep. Symptoms of RLS include unpleasant sensations, often described as creeping, crawling, tingling, or aching in the legs and the urge to move. The discomfort is typically relieved by moving the legs. Symptoms often increase when lying down or sitting for long periods. The condition can disrupt sleep, leading to insomnia and daytime fatigue. Research has shown a notable relationship between RLS and dementia, particularly in the elderly population. People with dementia, including Alzheimer’s disease, are at higher risk of experiencing RLS. This might be partly because RLS symptoms can be masked or misattributed to other behavioral changes associated with dementia. RLS frequently exacerbates sleep disturbances. Since poor sleep is a common issue in dementia, RLS can further impair sleep quality and exacerbate cognitive decline. RLS is often associated with iron deficiency. Low iron levels in the brain may contribute to the symptoms of RLS. Given that some dementia types are linked with alterations in brain iron metabolism, this relationship might influence the progression of dementia [70]. Certain medications used to manage dementia symptoms can worsen RLS. For instance, neuroleptics and other drugs with dopaminergic effects might aggravate RLS symptoms, thereby increasing nighttime agitation and worsening overall cognitive function. In individuals with dementia who cannot articulate their symptoms, RLS might be underdiagnosed. This misdiagnosis can lead to untreated RLS, which might contribute to behavioral disturbances and cognitive decline. Diagnosing RLS in dementia patients often requires careful evaluation, as traditional self-reporting may not be feasible. Tools like the Brief Inventory of Restless Legs (BIT-RL) can be helpful. Addressing RLS in dementia patients might involve managing iron deficiency, modifying medications that exacerbate symptoms, and employing treatments specifically for RLS. Improving sleep and reducing agitation through RLS management could enhance the quality of life for these patients. Overall, understanding and addressing RLS in the context of dementia is crucial for improving patient outcomes and managing behavioral and cognitive symptoms more effectively [71].

Exploring the characteristics and behaviors of older adults with Alzheimer’s disease and dementia (AD-D) who are diagnosed with restless legs syndrome (RLS) and experience caregiver-reported nighttime agitation, findings indicated that these patients had reduced sleep duration and increased fragmentation, with a significant percentage (79%) on medications that worsen RLS. Lower transferrin saturation (TS%) and shorter sleep duration were significantly associated with more frequent nighttime agitation, together accounting for 20% of the variance in these behaviors. The study, notable for its use of continuous, real-time observations by trained research assistants over two nights, is among the few to use the validated Brief Inventory of Restless Legs (BIT-RL) for diagnosing RLS in AD-D patients who cannot self-report symptoms. Although the study’s short observation period and potential limitations in excluding other disorders such as circadian rhythm disorders were noted, its rigorous methods support the findings. The research found that, despite no significant peripheral iron deficiency, lower TS% was linked with increased nighttime agitation, suggesting a potential new treatment avenue. The results imply that RLS may contribute to behavioral disturbances in dementia, and treating iron deficiency or modifying medication practices could alleviate symptoms. The BIT-RL appears feasible for use in both long-term care and home settings, providing valuable diagnostic information for clinicians. Future research should further investigate the relationship between RLS severity, nighttime agitation, and the use of medications that may exacerbate RLS [72, 73].

The severity of RLS directly correlates with the extent of these sleep disturbances. In mild cases, patients might experience occasional discomfort that minimally affects their sleep. However, as RLS symptoms become more severe, the disruptions to sleep become more frequent and pronounced. Patients may find it difficult to fall asleep or stay asleep, leading to chronic sleep deprivation. Over time, this lack of restorative sleep can contribute to the development of other issues, such as excessive daytime sleepiness, difficulty concentrating, and a general decline in daily functioning [74]. In addition to sleep disturbances, RLS is also associated with an increased risk of anxiety and depression. The chronic nature of the symptoms, combined with the persistent sleep disruption, can lead to significant emotional distress. Patients might become anxious about going to bed, knowing that their symptoms will likely worsen at night, which can create a vicious cycle of anxiety leading to further sleep disruption [75]. Moreover, the frustration and fatigue caused by ongoing sleep deprivation can contribute to the development of depressive symptoms, as patients struggle to manage the physical and emotional toll of the condition. The interplay between sleep disorders, anxiety, and depression in RLS patients can also impact cognitive function. Sleep is crucial for cognitive processes such as memory consolidation, problem-solving, and attention. When sleep is consistently disrupted, these cognitive functions can suffer. For instance, RLS patients might experience difficulties with memory recall, decision-making, and maintaining focus during the day. The severity of these cognitive issues often mirrors the severity of the sleep disruption, meaning that as RLS symptoms worsen, the impact on cognitive function becomes more pronounced. In summary, patients with RLS are at a heightened risk for sleep disorders, anxiety, and depression, all of which can have a compounding effect on their overall well-being. The severity of RLS symptoms plays a crucial role in the extent of these issues, with more severe cases leading to greater sleep disruption and, consequently, a more significant impact on cognitive function and mental health. Addressing these interconnected issues is essential for improving the quality of life for individuals with RLS [76, 77].

### Sundowning

**Figure 6:**
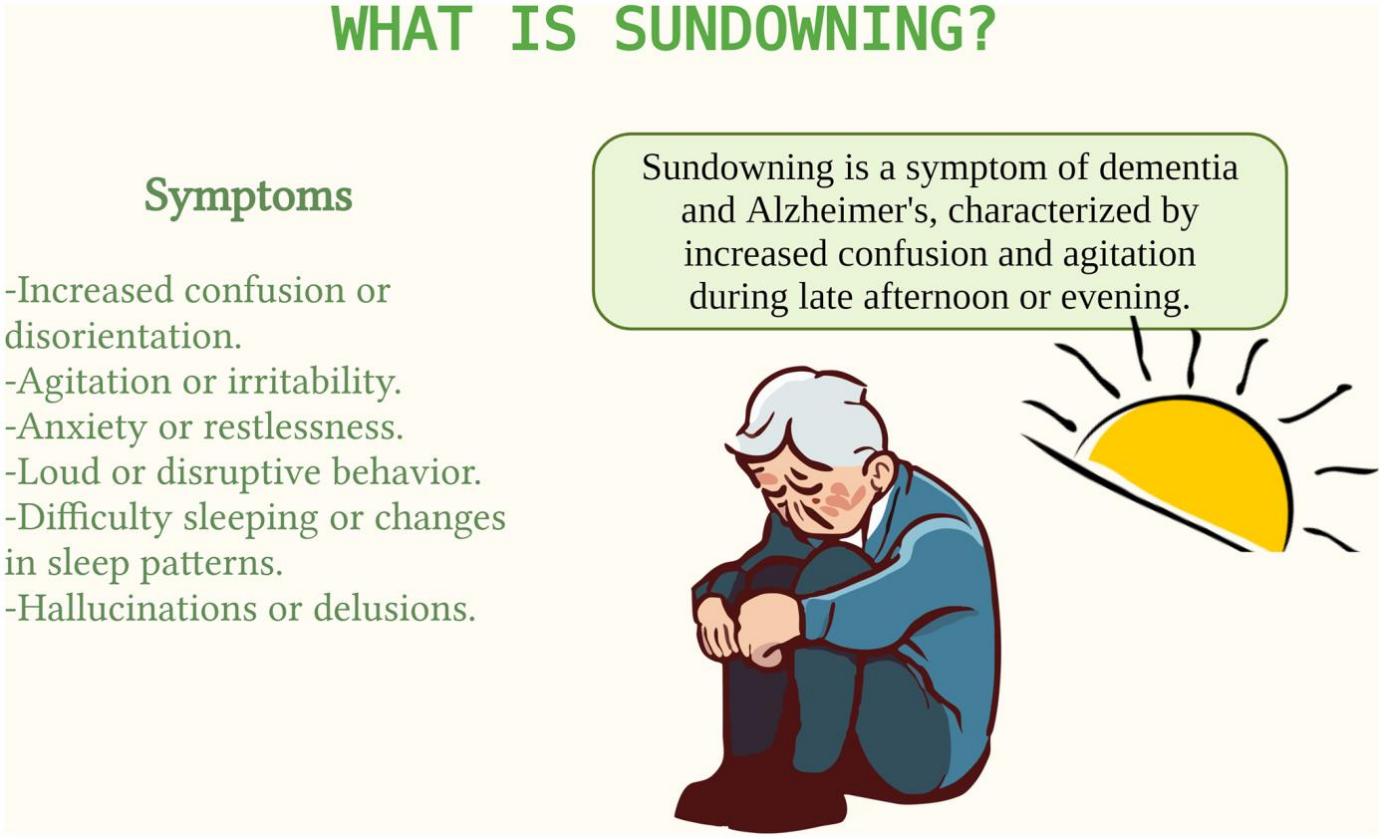
**Sundowning in dementia.** Sundowning is a phenomenon where individuals with dementia become more confused and agitated in the late afternoon and evening. It can be triggered by fatigue, reduced light exposure, and changes in routine. Managing sundowning is important for caregivers to enhance the comfort of those affected.

Sundowning is a term used to describe a pattern of increased agitation, confusion, and behavioral disturbances that occur in the late afternoon or evening in individuals with dementia. This phenomenon is particularly common in people with Alzheimer’s disease and other forms of dementia (Figure 6). Symptoms typically begin in the late afternoon or early evening and can worsen into the night. Increased confusion, agitation, restlessness, and irritability. Some individuals may also experience paranoia, hallucinations, or delusions during these times. Patients may have difficulty settling down, may become more demanding or combative, and may have disrupted sleep patterns [78].

The exact causes of sundowning are not fully understood, but several factors may contribute. Disruptions in the body’s internal clock can affect sleep-wake cycles and lead to increased confusion and agitation. Accumulated fatigue throughout the day can exacerbate symptoms as evening approaches. Reduced exposure to natural light in the evening can affect mood and cognitive function. Pain, discomfort, or untreated medical conditions can contribute to increased agitation. Changes in routine, unfamiliar surroundings, or overstimulation can trigger sundowning symptoms [79]. Managing sundowning involves a combination of environmental adjustments, behavioral strategies, and medical interventions. Establishing a consistent daily routine and maintaining a calm, comfortable environment can help reduce anxiety and confusion. Dim the lights in the evening and avoid stimulating activities close to bedtime. Encourage good sleep practices, such as having a regular bedtime routine and ensuring a restful sleeping environment. Encourage regular physical activity during the day to help reduce restlessness and improve sleep. Engaging in soothing and familiar activities in the late afternoon can provide comfort and reduce agitation. Regularly reviewing medications and medical conditions with a healthcare provider to ensure there are no underlying issues contributing to sundowning. Ensuring the environment is safe to prevent accidents during periods of increased confusion and restlessness. Sundowning is a challenging symptom for individuals with dementia and their caregivers. Understanding its patterns and implementing strategies to manage it can improve the quality of life for both patients and caregivers. Consulting with healthcare professionals for tailored approaches and treatments is also essential in managing sundowning effectively [80].

Sundowning is believed to be influenced by several interrelated factors like circadian rhythm disruption**,** neurochemical imbalances, environmental factors and cognitive decline. Dementia often disrupts the body’s internal clock, leading to irregular sleep-wake cycles. This disruption may worsen in the evening, contributing to sundowning symptoms. Alterations in neurotransmitters, such as melatonin and serotonin, which regulate mood and sleep, are common in dementia and may play a role in the onset of sundowning behaviors. Changes in lighting, reduced activity levels, and increased fatigue towards the end of the day can exacerbate confusion and agitation in dementia patients. As cognitive functions deteriorate, patients may struggle more with orientation and understanding their environment, leading to increased anxiety and behavioral issues in the evening. These mechanisms are interconnected, and the combination of biological, psychological, and environmental factors contributes to the occurrence of sundowning in dementia patients. Understanding these mechanisms can aid in developing more effective management strategies [81]. There are several mechanisms through which light therapy may help alleviate sundowning symptoms. Light therapy helps reset the body’s internal clock by exposing patients to bright light, particularly in the morning. This can stabilize sleep-wake cycles and reduce the likelihood of evening agitation and confusion. Exposure to appropriate light levels during the day can enhance nighttime sleep quality. Better sleep reduces overall fatigue and can mitigate the severity of sundowning behaviors. Light therapy may influence the production of melatonin, a hormone that regulates sleep. By increasing morning light exposure, the therapy can suppress daytime melatonin production, leading to a more natural rise in melatonin levels in the evening, promoting better sleep and reducing sundowning. Light therapy also serves as a form of environmental enrichment. By simulating natural daylight, it can reduce confusion and anxiety associated with dimly lit environments, which are often triggers for sundowning. Light therapy is a promising non-pharmacological intervention for managing sundowning in dementia patients, primarily through its effects on circadian regulation and sleep improvement [80].

## CONCLUSIONS AND FUTURE DIRECTIONS

Sleep disturbances are a common and particularly challenging aspect of dementia, profoundly affecting both individuals with the condition and their caregivers. These disturbances are not merely a symptom of dementia but are closely intertwined with the progression of the disease and its comorbidities. The complexity of sleep issues in dementia is underscored by their multifactorial nature, where neurobiological changes, comorbid medical conditions, psychological factors, and environmental influences all play critical roles. Neurodegenerative processes that characterize dementia, such as the accumulation of amyloid plaques and tau tangles, are known to disrupt the normal architecture of sleep. These disruptions can lead to fragmented sleep, reduced sleep efficiency, and alterations in the sleep-wake cycle, often manifesting as insomnia, nocturnal awakenings, and daytime sleepiness. Moreover, specific forms of dementia, such as Alzheimer’s disease, Lewy body dementia, and Parkinson’s disease dementia, are associated with unique sleep disturbances like REM sleep behavior disorder, sleep apnea, and restless legs syndrome, further complicating the clinical picture.

Comorbid medical conditions, which are common in the elderly population, exacerbate sleep problems in dementia patients. Conditions such as cardiovascular disease, chronic pain, depression, and anxiety not only contribute to sleep disturbances but are also linked to the worsening of cognitive function. The bidirectional relationship between sleep and these comorbidities highlights the importance of a holistic approach to managing dementia. For instance, untreated sleep apnea in a dementia patient can accelerate cognitive decline, while effective management of this condition can improve both sleep quality and cognitive outcomes. The impact of sleep disturbances extends beyond the patients themselves, significantly affecting caregivers. Caregivers of dementia patients with severe sleep disturbances often experience heightened stress, fatigue, and burnout, which can lead to poor mental health and a reduced ability to provide care. This situation creates a vicious cycle where caregiver distress exacerbates patient symptoms, further increasing the burden on caregivers. Therefore, addressing sleep issues is crucial not only for the well-being of dementia patients but also for their caregivers, who are integral to the care process [82].

Environmental factors and daily routines also play a critical role in sleep disturbances among dementia patients. Factors such as noise, light exposure, and irregular sleep schedules can exacerbate sleep problems. Conversely, interventions aimed at improving the sleep environment, such as reducing noise and maintaining a consistent sleep-wake schedule, can have a significant positive impact on sleep quality. Non-pharmacological interventions, including cognitive-behavioral therapy for insomnia (CBT-I), light therapy, and physical activity, have shown promise in managing sleep disturbances without the risks associated with pharmacological treatments. Given the complex interplay between sleep, dementia, and comorbidities, a multidisciplinary approach is essential in managing these patients. This approach should involve not only neurologists and geriatricians but also sleep specialists, psychiatrists, and primary care providers, ensuring that all aspects of the patient’s health are addressed. Early identification and treatment of sleep disturbances can slow the progression of cognitive decline, enhance the quality of life for dementia patients, and reduce the emotional and physical strain on caregivers.

In conclusion, sleep disturbances in dementia represent a critical but often overlooked aspect of care. Addressing these disturbances through a comprehensive and individualized approach can lead to significant improvements in patient outcomes and caregiver well-being. As the population ages and the prevalence of dementia rises, integrating sleep management into the standard care for dementia will become increasingly important in mitigating the broader impact of this devastating condition.
